# Perspective Space as a Model for Distance and Size Perception

**DOI:** 10.1177/2041669517735541

**Published:** 2017-11-29

**Authors:** Casper J. Erkelens

**Affiliations:** Experimental Psychology, Helmholtz Institute, Utrecht University, The Netherlands

**Keywords:** visual space, perceived distance, perceived size, physical space

## Abstract

In the literature, perspective space has been introduced as a model of visual space. Perspective space is grounded on the perspective nature of visual space during both binocular and monocular vision. A single parameter, that is, the distance of the vanishing point, transforms the geometry of physical space into that of perspective space. The perspective-space model predicts perceived angles, distances, and sizes. The model is compared with other models for distance and size perception. Perspective space predicts that perceived distance and size as a function of physical distance are described by hyperbolic functions. Alternatively, power functions have been widely used to describe perceived distance and size. Comparison of power and hyperbolic functions shows that both functions are equivalent within the range of distances that have been judged in experiments. Two models describing perceived distance on the ground plane appear to be equivalent with the perspective-space model too. The conclusion is that perspective space unifies a number of models of distance and size perception.

## Introduction

Physical space can be defined as the boundless three-dimensional extent in which objects have size, form, and position. Physical space is homogeneous and isotropic within the extent of human vision, implying that objects do not change in size or form under translation and rotation. Visual space is the extent that we, that is, human beings, perceive through vision. Visual space differs from physical space, especially at long viewing distances. It is neither homogeneous nor isotropic, implying that objects are perceived to change in size or form under translation and rotation. Perspective space has been proposed as a model of visual space (Erkelens, 2015a; Gilinsky, 1951; Hatfield, 2012). Gilinsky (1951) introduced the model to describe empirical data of distance and size perception. The single parameter of the model, that is, the distance of the vanishing point was inferred to be about 30 m or more. Erkelens (2015a) used perspective space to describe perspective angles, that is, angles perceived between parallel lines in physical space. Distances of the vanishing point inferred from perspective angles were shorter than 6 m. The large difference between the distances of vanishing points in the two studies suggests that the models of Gilinsky and Erkelens have different geometries. An alternative explanation is that perceived distances and angles cannot be described by a single perspective space. The purpose of this study is to investigate properties of perspective space in relation to distance and size perception and to compare the models of Gilinsky and Erkelens with each other and with other models of distance and size perception.

Research of distance and size perception has a long history (for a comprehensive review see Wagner, 2006). The extensive literature on the topic presents a plethora of experimental results, which together do not seem to go well with a specific geometry of visual space. Results depended so heavily on methods, conditions, and instructions that researchers even repudiated the concept of visual space altogether (Cuijpers, Kappers, & Koenderink, 2002). Wagner (2006) championed the idea, less remote from the intuitive notion of a visual space, that we should see visual space as a family of spaces whose individual geometries differ from each other depending on experimental conditions and mental shifts in the meaning of size and distance. This study will show that perspective space is such a family of spaces. Perspective space will prove to be an attractive model for distance and size perception because it fits well to many experimental results and unifies a number of existing models. Another attractive property of perspective space is that it matches both physical space and pictures in a natural and simple way.

## Erkelens’ Model of Perspective Space

**Figure 1.**
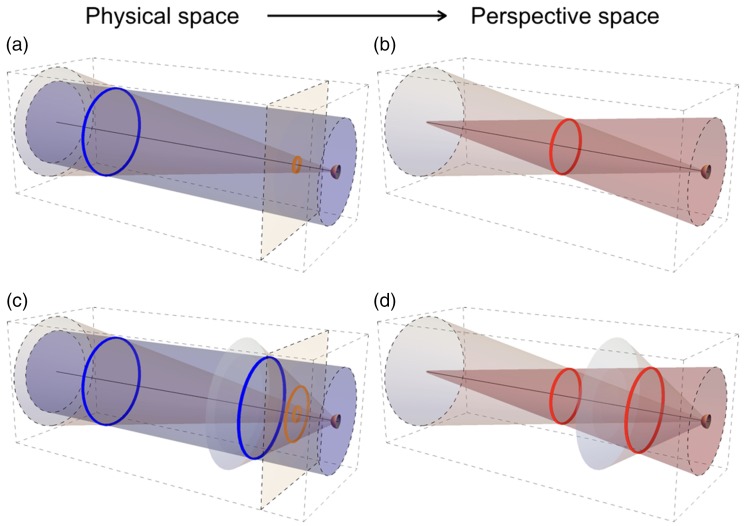
Sizes and distances of rings in physical and perspective space. (a) One ring in physical space. The observer (half sphere at the right side) fixates the center of the ring (blue). The black line indicates the (binocular or monocular) viewing direction. The ring defines a set of directions (forming the cone with its vertex at the observer). The ring also defines a set of lines parallel to the viewing direction, indicating physical trajectories if the ring would move in the viewing direction. The parallel lines together form a cylinder, projecting a circle the size of the ring in the plane of the observer. The orange plane orthogonal to the viewing direction contains the projection (orange) of the ring on a two-dimensional planar surface. (b) The ring of (a) in perspective space. The directional cone is identical to that in physical space. The parallels in physical space are converted to lines converging to a vanishing point in perspective space, indicating trajectories if the ring would move in the viewing direction. Together, the converging lines form a cone having its vertex at the vanishing point. Intersection between the two cones forms the ring (red) in perspective space. (c) Two identical rings at different distances in physical space. The two rings (blue) project to two concentric rings (orange) in the picture plane, indicating their relative size on the retina. (d) The two rings of (c) in perspective space. Size ratio of the rings (red) depends on the distance of the vanishing point and lies in between size ratios in the picture plane (orange) and physical space (blue) for positive finite vanishing distances.

Perspective space is the collective name for spaces that differ from each other by the value of a single parameter, that is, the distance of the vanishing point. Figure 1 shows objects, rings in this example, in physical space (Figure 1(a) and (c)) and how their sizes, distances, and directions are transformed in perspective space (Figure 1(b) and (d)). Object size is independent of distance in physical space (Figure 1(c)) but not in perspective space (Figure 1(d)). Perspective space is defined relative to the position and viewing direction of an observer. The distance of its vanishing point characterizes a certain perspective space. Generally, the distance is finite meaning that perspective space is bounded in depth. The family of perspective spaces includes two spaces whose geometries are equivalent to spaces in the physical world. The analogue spaces are physical space itself and the projection of physical space on a flat surface orthogonal to the viewing direction, that is, the picture plane (Figure 1(a) and (c)), a planar representation of the retinal image. Distance of the vanishing point is infinite for physical space and zero for the picture plane. Positions of objects in perspective space are best expressed in terms of a two-dimensional direction relative to the viewing direction and a one-dimensional depth relative to the position of the observer. In perspective space, depth depends on distance of the vanishing point but direction does not, implying that directions of objects are identical in all perspective spaces, including physical space and the picture plane.

**Figure 2.**
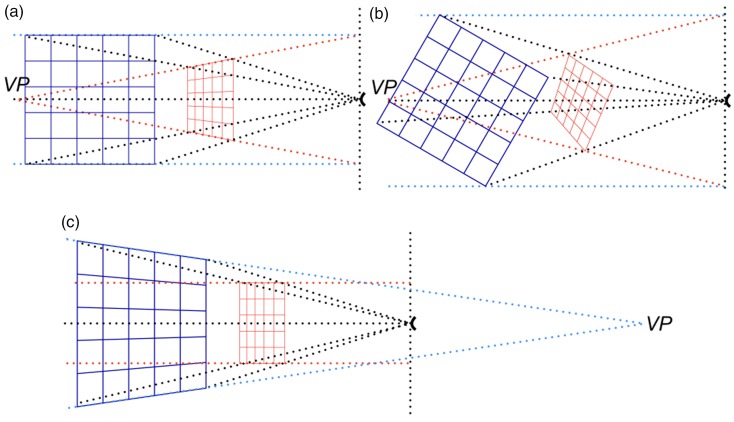
Transformations between physical and perspective shapes. (a) A two-dimensional grid in physical space (blue) is located at a certain distance from the observer (indicated by arcs at the right side). Each point of the grid defines a direction (the black dotted lines are examples). Each point also defines a line parallel to the viewing direction (blue dotted lines) intersecting with the frontal plane of the observer. The parallels in physical space converge to the vanishing point *VP* in perspective space (red dotted lines). Intersections between directions and vanishing lines define points of the grid in perspective space. Together the intersections form a deformed grid (red). Transformation from physical to perspective space affects shape, size and distance of the grid. A physical grid is shown whose lines are parallel or orthogonal to the viewing direction. (b) The same physical grid is shown but rotated clockwise by 30° about its center. Note that the computed perspective grids of (a) and (b) are not rotated versions of each other. (c) The parallels in perspective space converge to a vanishing point *VP* in physical space lying behind the observer.

Perspective space is Euclidean, meaning that the metric is the straight-line distance and angles of triangles add up to 180° (Erkelens, 2015c). A property of perspective space is that straight lines in one perspective space constitute straight lines in any other perspective space (Figure 2). As a consequence, line pieces aligned in physical space remain aligned in perspective space (Erkelens, 2015c). This property has been shown experimentally for visual space (Cuijpers et al., 2002). Another property of perspective space is that parallel lines in one space generally transfer to converging or diverging lines in other spaces. Thus, parallelism is not preserved. Parallel lines in frontal planes are the exception. Such lines remain parallel in all perspective spaces. Experimentally, parallelism was found not preserved in visual perception for lines having orientations in depth (Cuijpers, Kappers, & Koenderink, 2000). Figure 2(a) shows that lines in physical space running parallel to the viewing direction converge in perspective space to a vanishing point *VP* lying in front of the observer. Conversely, lines in perspective space parallel to the viewing direction converge in physical space to a vanishing point *VP* lying behind the observer (Figure 2(c)). The famous parallel alleys of Hillebrand (1902) and Blumenfeld (1913) are examples of parallel lines in visual space that are described by the model of perspective space (Erkelens, 2015c). The distance alleys of Blumenfeld (1913) are explained by perspective space in combination with the size-distance-invariance hypothesis (Epstein, 1963). Figure 2(b) shows parallel lines in physical space that have an orientation in depth different from the viewing direction. These lines converge to vanishing points of other perspective spaces. These perspective spaces are defined by viewing directions running parallel to the lines of the grids in physical space.

## Distance and Size Perception

**Figure 3.**
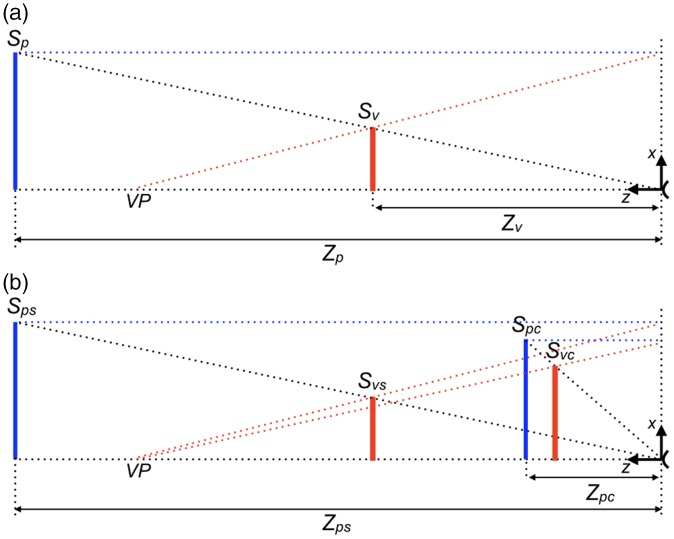
Geometries for distance and size judgments. (a) A line in physical space (blue) of size *S_p_* is located at distance *Z_p_* from the observer (arc at the right side). In perspective space, as a model for visual space, the line (red) gets size *S_v_* and distance *Z_v_* if the vanishing point is placed at distance *VP*. (b) A standard line in physical space of fixed size *S_ps_* is located at a variable distance *Z_ps_* from the observer. A comparison line in physical space of adjustable size *S_pc_* is located at a fixed distance *Z_pc_* from the observer. In visual space, the lines have sizes *S_vs_* and *S_vc_*, respectively*,* if the vanishing point is at distance *VP*. The vertical, dotted lines on the right side of the graphs represent the plane of the observer orthogonal to the viewing direction.

The model of perspective space makes predictions for distance and size perception. Since direction and distance behave differently in perspective space, use of a polar coordinate system would be appropriate. However, perceived distances and sizes are usually expressed in meters rather than degrees. Therefore, it is convenient to use a Cartesian coordinate system having its origin at the observer and the *z*-axis along the viewing direction (Figure 3). Furthermore, Cartesian coordinates are helpful in comparing derived equations directly to equations presented in the literature.

Figure 3(a) shows in one graph the right half of the cross-sections along the *z*-axes of the physical and perspective spaces shown in Figure 1(a) and (b). Since perspective space is used as a model of visual space, the term visual will be used as a replacement of the term perspective.

The relationship between distance in visual and physical space is given by

(1) $$\frac{Z_{v}}{Z_{p}}=\frac{\mathrm{VP}}{Z_{p}+\mathrm{VP}}$$

Since *S_p_* and *S_v_* define similar triangles relative to the observer, sizes have the same relationship as distances

(2) $$\frac{S_{v}}{S_{p}}=\frac{\mathrm{VP}}{Z_{p}+\mathrm{VP}}$$

The two equations show that perceived distance and size depend both on physical distance and the vanishing distance of visual space. Although the relationship between physical and visual size is simple, it cannot directly be applied to fit experimental results presented in the literature. Usually, observers matched sizes of two objects. One object, called the standard, was fixed of size and placed at various distances. The other object, called the comparison, was placed at a fixed distance and adjustable of size. Figure 3(b) shows the geometry for a set of standard and comparison objects. The general relationship between the physical and visual sizes of the two objects is given by

(3) $$\frac{S_{\mathrm{pc}}}{S_{\mathrm{ps}}}=\frac{S_{\mathrm{vc}}}{S_{\mathrm{vs}}}\frac{Z_{\mathrm{pc}}+\mathrm{VP}}{Z_{\mathrm{ps}}+\mathrm{VP}}$$

Equations (1) and (3) are derived in the Appendix.

Matching perceived sizes in a typical experiment means that observers are asked to set *S_v__c_* equal to *S_v__s_*, reducing Equation (3) to

(4) $$\frac{S_{\mathrm{pc}}}{S_{\mathrm{ps}}}=\frac{Z_{\mathrm{pc}}+\mathrm{VP}}{Z_{\mathrm{ps}}+\mathrm{VP}}$$

**Figure 4.**
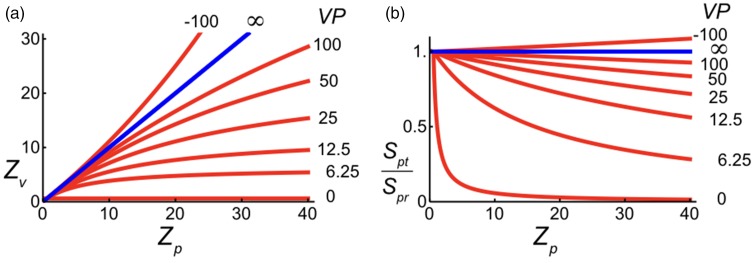
Effect of vanishing distance on perception of distance and size. (a) Perceived distance *Z_v_* computed as a function of physical distance *Z_p_* for a number of distances of the vanishing point *VP*. (b) Size of the comparison stimulus *S_pc_* relative to size of the standard *S_ps_* computed as a function of physical distance *Z_p_* for a number of distances of the vanishing point *VP*. *Z_v_*, *Z_p_*, and *VP* are distances expressed in meters. Although both physical space and perspective space are rectilinear (flat), the perspective transformation of the noninfinite vanishing point implies that the relationship between distances in the two spaces represented in these plots is curvilinear.

Figure 4(a) shows relationships between visual and physical distances as expressed by Equation (1). The graph shows three classes of relationships. If *VP* is positive, visual distance is an underestimation of physical distance and becomes equal to *VP* for objects at infinity. In other words, visual space is a bounded space. If *VP* is infinite (blue line), visual and physical distances are equal. Then, visual space is unbounded. If *VP* is negative, observers overestimate physical distances. Negative *VP*s are associated with inverted perspective (Arnheim, 1972), also called reverse perspective (Derksen, 1999; Wade & Hughes, 1999), or occasionally, Byzantine perspective (Deregowski, Parker, & Massironi, 1994). If *VP* is negative, parallel lines in physical space are perceived to diverge rather than converge with distance. Negative *VP*s are discussed later in relation to instructions that have been given to observers in size and distance judgment tasks.

Figure 4(b) shows relationships for perceived size as described by Equation (4). The graph shows ratios for two equally large perceived stimulus sizes positioned at different distances. The horizontal line (blue) for which *VP* is infinite shows the law of size constancy indicating that perceived size is independent of distance. Perceived size decreases with distance for positive *VP*s. This phenomenon is called underconstancy of size. Decrease in size with distance is fully determined by stimulus size in the picture or on the retina, respectively, if *VP* is zero. The negative *VP* is associated with overestimating size with increasing distance.

## Other Models of Distance and Size Perception

### Gilinsky’s Model

Equations (1) and (2), describing the relationship between visual and physical distances and sizes, are identical to the relationships derived by Gilinsky (1951). This means that the models of Gilinsky and Erkelens have the same geometry. According to Gilinsky herself, computations were mainly inspired by Luneburg’s theory of a curved visual space (1947, 1950). Gilinsky (1951, p. 460) stated,

the two formulas [for distance and size] are rigorously derived from the basic metric of visual space as established mathematically (for binocular vision) by Luneburg (1947, 1950). Second, the same two formulas are mathematically derived (somewhat less rigorously but without restriction to binocular vision) from the known principles of visual perspective. Finally, the same two formulas are derived by a simple inductive method of mathematical composition for the two boundary laws of size constancy and retinal image (visual angle). All three methods of derivation yield the identical pair of formulas to express a unifying law of visual space perception.

As Fry (1952) pointed out, Gilinsky (1951) made substitutions in Luneburg’s equations, which turned Luneburg’s essentially non-Euclidean metric into a Euclidean metric. Thus, Gilinsky (1951) used a Euclidean metric and described a flat rather than curved space for the domains of monocular as well as binocular vision. Gilinsky’s equation has been very successful in describing considerable amounts of experimental data (Fry, 1952). Nevertheless, Baird and Wagner (1991) dismissed Gilinsky’s equation for distance perception because the computed equation could not describe overestimation of distance as was observed in a number of experimental studies.

Equation (4) is mathematically equivalent to the equation Gilinsky (1951) derived for size ratios. However, interpretation and usefulness are different. Gilinsky (1951) computed size ratios by assuming a distance, called the “normal” viewing distance, at which perceived size was equal to physical size, which she called the “true” size. Thus, in size judgments between comparison and standard stimuli, distance of the comparison stimulus was limited to the one believed to be “normal”. Equation (4) does not have this limitation because it describes the ratio between two physical sizes, which are perceived as equally large. Both physical sizes can be positioned at any distance from the observer. Equation (4) is a special case of Equation (3). Equation (3), describing the general relationship between physical and perceived sizes, is also valid for conditions in which physical sizes are perceived differently from each other. For instance, it can be used in a task where the size of one object is judged as being twice the size of another.

### Ooi and He’s Model

**Figure 5.**
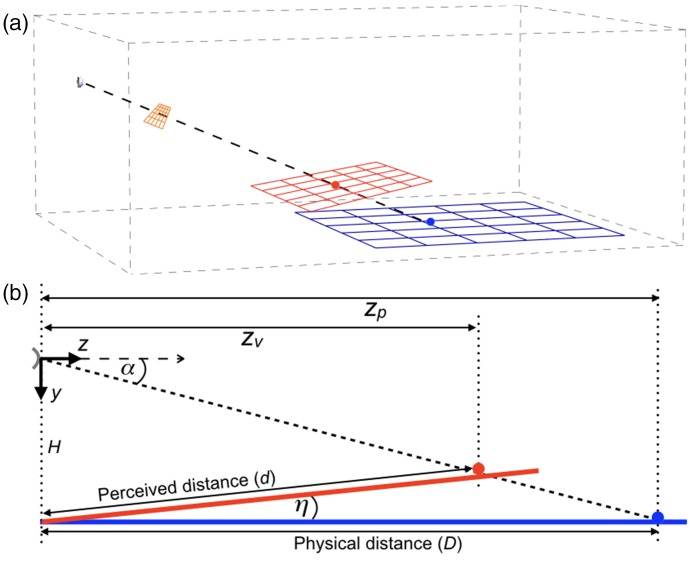
Relationship between perceived and physical distance on the ground plane. (a) Geometry of grids is according to the perspective-space model. The grid (blue) on the ground in physical space has an equivalent grid (red) in visual space, whose distance of the vanishing point is finite. The orange grid represents the observer’s proximal image, that is, the projection of the physical grid onto a plane orthogonal to the viewing direction (dashed line). (b) The vertical cross-section along the *z*-axis of (a) shows the geometry for an observer at height H above the ground, judging the distance from his feet to an object on the ground. Geometry and symbols are identical to those used by Ooi and He (2007). Viewing direction is along the *z*-axis. The blue and red points indicate associated locations in physical space and visual space.

Many studies have reported that judged distance is influenced by ground surface information (Bian, Braunstein, & Andersen, 2005; Feria, Braunstein, & Andersen, 2003; He & Ooi, 2000; He, Wu, Ooi, Yarbrough, & Wu, 2004; Madison, Thompson, Kersten, Shirley, & Smits, 2001; Meng & Sedgwick, 2001, 2002; Ni, Braunstein, & Andersen, 2004; Ooi, Wu, & He, 2001, 2006; Philbeck & Loomis, 1997; Sinai, Ooi, & He, 1998; Wu, Ooi, & He, 2004). Ooi and He (2007) took errors in perceived slant of the ground surface as the starting point for deriving a distance equation. The equation reads

(5) $$d=\frac{\mathrm{DH}/\text{sin}\eta }{H\text{cos}\eta /\text{sin}\eta +D}$$

where *d* is perceived distance, *D* is physical distance, *H* is height of the eye above the ground, and *η* is perceived slant of the ground surface. The authors showed that their ground-based equation took the same form as Gilinsky’s equation if slant error was small. Difference between slants of planes in physical and visual space is a characteristic property of the perspective-space model if vanishing distances are finite (Erkelens, 2015c). Figure 5(a) shows computed grids in physical space, visual space and the picture plane according the perspective-space model. The grid on the ground surface in physical spaces is slanted towards the observer in visual space if the distance of its vanishing point is finite. Perceived slant depends on vanishing distance. At one extreme, the visual grid coincides with the physical grid if the distance is infinite. At the other extreme, the visual grid becomes oriented orthogonal to the viewing direction if the distance is zero. The equation for perceived distance of objects on the ground plane derived by Ooi and He (2007) is almost identical to the one derived from the model of perspective space. The equation is derived here for the geometry presented by Ooi and He (2007), in which the observer views along the *z*-axis (Figure 5(b)). In the Appendix, the equation is derived for an observer fixating the object on the ground. The equations are identical in the two viewing conditions.

With $Z_{v}=d\text{cos}\eta$ and $Z_{p}=D$, Equation (1) reads

(6) $$\frac{d\text{cos}\eta }{D}=\frac{\mathrm{VP}}{D+\mathrm{VP}}$$

Because VP=H/sinη, Equation (6) can be rewritten as follows

(7) $$d=\frac{\mathrm{DH}/\text{sin}\eta }{H\text{cos}\eta /\text{sin}\eta +D\text{cos}\eta }$$

Equation (7) is almost identical to Equation (5) proposed by Ooi and He (2007). Term Dcosη is different, it replaces the term *D* in Ooi and He’s equation. However, for angles *η* < 4°, as have been measured by Ooi and He (2007), the terms differ less than 0.5%.

### Wagner’s Models

**Figure 6.**
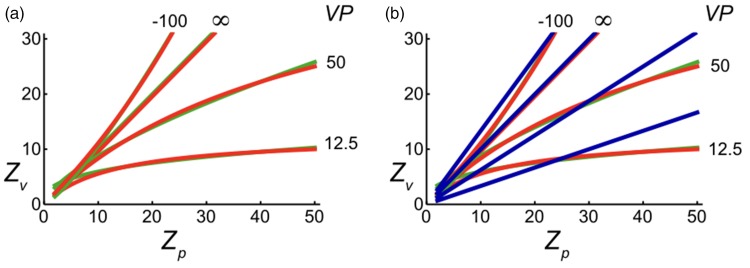
Comparison of distance functions. (a) Hyperbolic functions (red) are fitted to power functions (green) predicting relationships between visual (*Z_v_*) and physical (*Z_p_*) distance. Distances of the vanishing point (*VP*) specify the hyperbolic functions. (b) Linear contraction functions (blue) are fitted to power (green) and hyperbolic functions (red). *Z_v_*, *Z_p_*, and *VP* are distances expressed in meters.

Several investigators of distance judgments have proposed a power function for the relationship between perceived and physical distance (Baird & Wagner, 1991; Da Silva, 1985; Haber, 1985; Toye, 1986; Wagner, 1985, 2006; Wiest & Bell, 1985). The relationship can be written as $d=\lambda D^{\gamma }$. The power function has two parameters, namely scaling factor *λ* and exponent *γ*. Wagner (2006) fitted power functions to a great number of data from the literature. Across the board, the fits were very good. Wagner (2006) did not fit hyperbolic functions to the same set of data. Hyperbolic functions have only one parameter, the vanishing distance *VP*. To investigate differences between the two functions, hyperbolic functions were fitted to power functions presented by Wagner (2006). Fits were made for physical distances between 2 m and 50 m, distances relevant for the reported judgments (Figure 6(a)). Fits were made to the full range of power functions that described the experimental distance judgments. The area between the hyperbolic and power function fits as a percentage of the area between the power function fit and the *x*-axis was used as a measure for the difference between the two fits. Differences were smaller than 2% for hyperbolic functions having *VP*s larger than 20 m. Differences were larger for hyperbolic functions with smaller *VP*s mainly due to poor fits at the very short distances. Considering the variability in distance judgments, hyperbolic functions would have fit the experimental data about equally well as did the power functions. The hyperbolic and power functions become very different from each other at very far distances because perceived distance is bounded for hyperbolic functions but not for power functions. The fact that the perceived distance of extremely far objects, such as the moon, is noninfinite implies that visual space has a bounded extent. This property of visual space argues against using power functions for describing perceived distances. An argument in favor of hyperbolic functions is that these functions follow directly from a model of visual space, namely perspective space. There is yet no model of visual space that predicts power functions.

Another model of Wagner (1985, 2006) describes distances between visible stakes in three-dimensional space. Analysis of judgments of distances between stakes that were randomly placed in two- and three-dimensional spaces showed that physical distances were seen more than twice as large in frontal orientations as these were in in-depth orientations (Wagner, 1985). This result led to formulation of the vector contraction model of visual space. According to this model, the component of physical space frontal to the observer is unchanged in visual space, but the in-depth component is contracted. According to the model, frontally oriented sizes obey perfect size constancy. In-depth oriented sizes are contracted linearly as a function of distance. This implies that the model is not compatible with power and hyperbolic functions of distance. Figure 6(b) shows that linear functions only match power and hyperbolic functions for contraction factors near one, and thus, for visual spaces that closely match physical space.

### Foley’s Model

**Figure 7.**
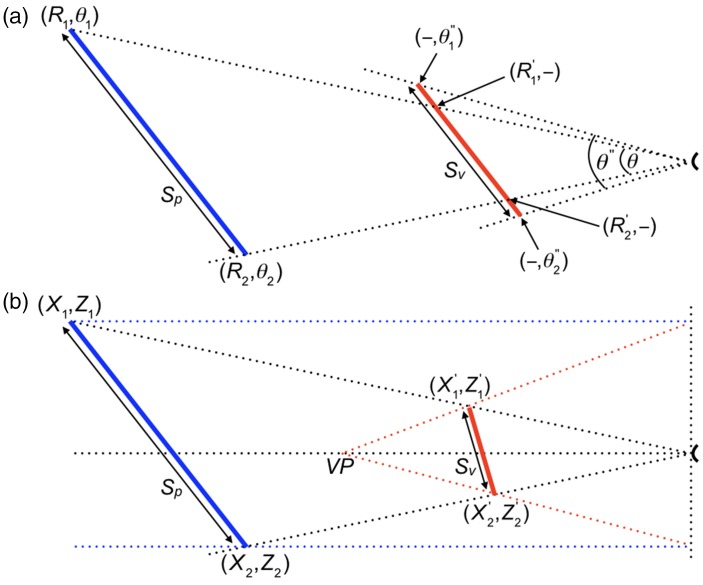
Comparison of the perspective-space model with Foley’s model. (a) According to Foley’s model, a physical line (blue) of size *S_p_*, whose ends are located at distances *R_1_* and *R_2_*, is perceived as a line (red) of size *S_v_* having the ends at distances *R′_1_* and *R′_2_*. (b) The same physical line and how it is perceived according to the perspective-space model.

**Figure 8.**
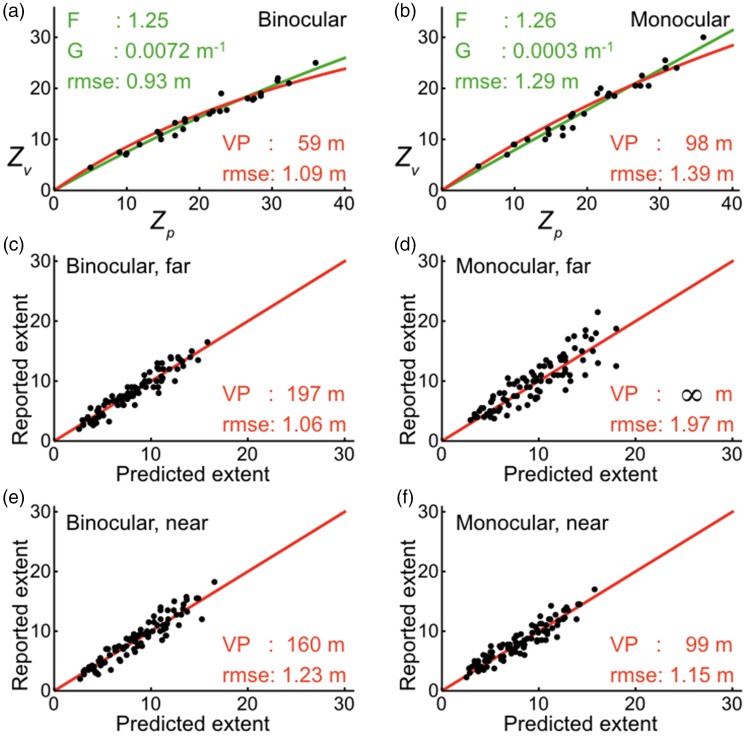
Model predictions for distance and extent. (a) Perceived distance (*Z_v_*) measured during binocular viewing of stakes positioned at various physical distances (*Z_v_*) to the observer. Data are from Foley et al. (2004). Lines are fits by the models of Foley (green) and perspective space (red). (b) Perceived distance measured during binocular viewing. (c, d, e, and f) Reported extents in four experimental conditions are plotted against extents predicted by the perspective space model. Red lines indicate perfect predictions.

Foley, Ribeiro-Filho, and Da Silva (2004) also investigated perceived distances between stakes in three-dimensional space, which the authors called perceived extents. Foley et al. (2004) proposed a model in which perceived extent is proportional to the product of magnified image size and perceived distance (Figure 7(a)). The computations of extents were based on three equations with in total four free parameters. Foley et al. (2004) computed perceived extent as $S_{v}=\sqrt{(R_{1}^{'}{)}^{2}+(R_{2}^{'}{)}^{2}-2R_{1}^{'}R_{2}^{'}\text{cos}\theta^{\prime \prime }}$, where $\theta^{\prime \prime }=\theta +Q\theta^{P}$ and $R^{'}=R/(F+GR)$. Magnified image size $\theta^{\prime \prime }$ was obtained from image size θ by adding a term with the two free parameters *Q* and *P*. Perceived egocentric distance *R′* was obtained from physical distance *R* by the expression with the two parameters *F* and *G*. The expression for *R′* resembles somewhat that for perceived distance in perspective space as described by Equation (1). However, Equation (1) relates perceived to physical distance with help of only one parameter. Foley et al. (2004) used two parameters (*F* and *G*) for relating perceived to physical distances and another two parameters (*Q* and *P)* for relating extents to image sizes. To compare fits to data of perceived distances and extents by Foley’s model with fits by the model of perspective space, perceived positions (*X’*, *Z’*) according to the model of perspective space were computed from the physical positions (*X*, *Z*) of the stakes by applying Equations (1) and (2). Extents *S_p_* and *S_v_* were computed as Euclidean distances (Figure 7(b)). Foley et al. (2004) recorded the physical coordinates of the 14 stakes used in their experiments in a table as (*X*, *Z*) coordinates. In another table, the authors recorded all the measured median extents in four groups of 91 data points, namely, separately for binocular and monocular viewing and for viewing at far and near distances. The perceived egocentric distances of the stakes are shown as a function of their physical distances for binocular viewing in Figure 8(a) and for monocular viewing in Figure 8(b). The data were fit by Foley’s distance function with the parameters *F* and *G*, and by the distance function of perspective space with parameter *VP*. The current values computed for *F* and *G* and root mean square errors (RMSE) are identical to those given by Foley et al. (2004). Both models provided good fits to the data. The slightly smaller root mean square errors for Foley’s model were to be expected because the model contains two free parameters and the perspective-space model just one. Adjusted *R*^2^ values, as a goodness-of-fit measure for the two nonlinear models, were hardly different from each other (Foley: 0.996 (binocular), 0.994 (monocular); perspective: 0.994 (binocular), 0.992 (monocular)).

Extents computed by the perspective space model were compared with reported extents for a range of distances of the vanishing point (*VP*). Figure 8(c) to (f) shows the results for values of *VP* that produced the lowest root mean square errors (RMSEs). On average, the RMSEs are slightly larger than those resulting from Foley’s model (Foley et al. 2004). For perceived distance during binocular viewing, fits of the perspective model were best for *VP* having a distance of 59 m. Optimal *VP*s were about three times as large for perceived extents under the same viewing condition. During monocular viewing, best fits of the distance data were computed for a *VP* of 98 m. The optimal *VP* was about equal for near extents. For far extents, fits of the perspective model were somewhat poorer. Best fits were obtained for *VP*s indistinguishable from infinity.

## Discussion

Perspective space is not a neurobiological model of visual space. It does not explain or even suggest how visual space is constructed from retinal images and neural processes. Instead, perspective space describes how geometric quantities in visual space relate to those in physical space and pictures. The model is based on two assumptions about visual space. One assumption is that visual space is Euclidean, implying that geodesics are straight lines. Looking at a straight railway line, road, or tube oriented in depth shows that preserved straightness is a reasonable assumption for far and central vision (Erkelens, 2015a, 2015b). Visual space being Euclidean also implies that line pieces aligned in physical space remain aligned in visual space (Cuijpers et al., 2002). The second assumption is that visual directions are identical in physical and visual space. Evidence for identical directions comes from aiming devices and eye movements. In the event of differences between physical and visual directions, these would occur as offsets or magnifications of the visual field relative to the physical field. Offsets are highly improbable because all kinds of aiming devices would be useless otherwise. Magnifications are improbable too because voluntary saccadic eye movements made between continuously visible targets are highly accurate relative to the required retinal angles (Collewijn, Erkelens, & Steinman, 1988; Erkelens, Steinman, & Collewijn, 1989). One could argue that eye movements and other motor actions operate on stimuli in physical space and do not affect visual space. However, convincing arguments in the empirical sciences support the view that perception of the external world is scaled by action-specific constraints (Barsalou, 2008; Bourgeois & Coello, 2012; Fajen, 2005; Gallese, 2007; Witt & Proffitt, 2008).

Fitting the perspective-space model to perceived angles, distances, and sizes resulted in a wide range of inferred vanishing-point distances. Although data come from different studies and observers, it is hard to imagine that visual space defined by a single vanishing-point distance can describe all the judgments of individual observers. To illustrate this, data from individual observers showed that eye height affects judgments of distance (Ooi & He, 2007) and in-depth oriented angles (Erkelens, 2015a). Comparison of different studies suggests that distances of vanishing points also depend on the attribute that is judged. Distances of vanishing points computed from judgments of in-depth oriented angles are shorter than 6 m (Erkelens, 2015a). Distances computed from the parallel-alley data of Blumenfeld (1913) were even shorter than 1 m, probably because of the extremely small eye height at which the stimuli were viewed. Vanishing distances computed from distance judgments (Foley et al., 2004; Gilinsky, 1951) range from about 30 m to 100 m. Vanishing distances computed from size judgments made in the same studies range from about 100 m to infinity. The vanishing point is a theoretical attribute of perspective space. It is questionable whether observers can judge its distance. The wide range of inferred distances of vanishing points suggests that visual space is best described by a perspective space whose depth depends on condition and attribute. Apparently, observers are insensitive to the fact that different attributes of depth belong to different perspective spaces. The insensitivity is convincingly illustrated by a great number of perspective paintings. Laymen as well as experts of perspective are not aware of inconsistencies between in-depth oriented angles and distances in many high-quality paintings of famous artists (Erkelens, 2016).

### Comparison With Competing Models

**Figure 9.**
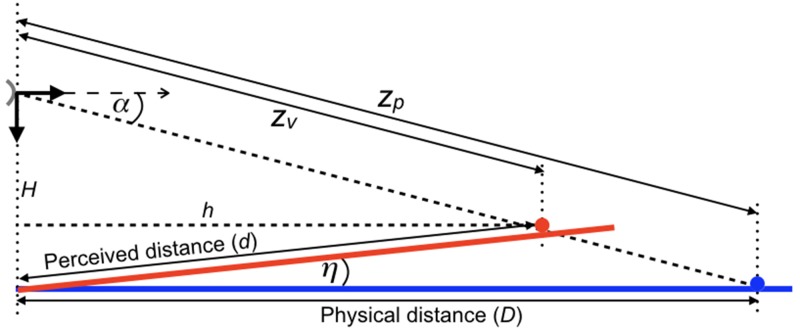
Relationship between perceived and physical distance. The observer at height H above the ground judges the distance from his feet to an object on the ground plane. Symbols are identical to those used in Figure 5(b). Viewing is in the direction of the object (blue) on the ground.

The perspective-space model has been compared with five models of distance and size perception. The first model was the mathematical model of Gilinsky (1951). Although based on different principles, equations for distance and size derived by Gilinsky (1951) are equivalent to those given by the perspective-space model. Advantage of the perspective-space model is its wider applicability and greater simplicity, giving analytical solutions for perceived distances, sizes, and angles. The second model was that of Ooi and He (2007) who proposed their model to describe a particular phenomenon, namely, foreshortening of distance on the ground plane. Ooi and He’s model describes perceived distances of objects on the ground relative to the feet of the observer. Computations of perceived distance require estimates of eye height and another perceptual parameter, namely, the perceived inclination of the ground plane. The almost identical equation given by the perceptual-space model shows that the experimental results of Ooi and He (2007) may reflect perceived and physical distances of objects (*Z_v_* and *Z_p_* in Figure 9) relative to the viewing point of the observer, that is, the eye or the head. Differences between both models are too small to decide which model best describes the data of Ooi and He (2007).Li, and Durgin (2012) proposed an alternative hypothesis, the angular expansion hypothesis. The hypothesis, assuming exaggerations in visual angle, was also used to describe perceived foreshortening of distance on the ground plane measured by Ooi and He (2007). The hypothesis was compared with the hypothesis of Ooi and He (2007), which they called the intrinsic bias hypothesis. Models based on each of the two hypotheses described the data equally well. Li and Durgin (2012), however, claimed more general usefulness for their hypothesis. The current computations show that the models of perspective space, Ooi and He, and Li and Durgin can be regarded as equivalent models for distance perception of objects on the ground plane. The third model was the power-function model for perceived distance proposed by Baird and Wagner (1991) and used in many studies. Differences between power and hyperbolic functions of the perspective-space model were very small over the entire range of distances in which judgments have been made. It is reasonable to conclude that both functions are equivalent in describing perceived distance. The fourth model was the vector-contraction model of visual space (Wagner, 1985). This model was developed to describe judgments of distances between randomly positioned stakes. Comparison with hyperbolic and power functions showed that extending the model to perceived distances along visual directions will give results that are incompatible with all the other models. Conclusion is that the contraction model of visual space may fit a particular purpose but cannot be a generic model of visual space. The fifth model was Foley’s model. Foley et al. (2004) proposed a model whose principal assumption was that, in the computation of perceived extent, the physical angle signal undergoes a magnifying transformation (Figure 7(a)).Figure 8 shows that the results of Foley et al.’s (2004) for egocentric distance and exocentric extent are described by the perspective-space model, distance and extent requiring different distances of the vanishing point. The models of Foley and perspective space have in common that distances and extents are not described by the same parameters. The perspective-space model is simpler and more generic in that it includes the description of perceived angles.

### The Role of Instructions in Distance and Size Perception

In a previous study, I argued that we have representations of both visual and physical space at our disposal (Erkelens, 2015a). For example, we see on the one hand that a road narrows in front of us but on the other hand we are confident that it does not. The same holds for size. We see that an approaching car becomes larger but at the same time are aware that its size stays the same. Our representation of physical space does not result from vision alone but also from other senses and motor interaction with the physical environment. For a yet unknown reason, our representations of visual and physical space do not merge into a single representation. The different representations give human beings the possibility to answer questions about spatial relationships in several ways. The hypothesis deviates from the view of many researchers who assumed that spatial judgments made under different instructions reflect properties of a single space. Famous are the parallel and distance alleys, initially measured by Hillebrand (1902) and Blumenfeld (1913). The alleys led to the concept of curved visual space (Luneburg, 1947, 1950). Results were confirmed and extended by many studies (Battro, di Pierro Netto, & Rozenstraten, 1976; Hardy, Rand, & Rittler, 1951; Indow, Inoue, & Matsushima, 1962; Luneburg, 1950; Roberts & Suppes, 1967; Shipley, 1957; Yamazaki, 1987; Zage, 1980; Zajaczkowska, 1956). The studies concluded that visual space is curved although a few authors challenged its hyperbolic nature. Later studies reported conflicting results but persevered in constructing curved visual spaces (Cuijpers, Kappers, & Koenderink, 2001; Cuijpers et al., 2000, 2002; Higashiyama, 1984; Indow & Watanabe, 1984a, 1984b; Koenderink, van Doorn, Kappers, Doumen, & Todd, 2008; Koenderink, van Doorn, Kappers, & Todd, 2002; Koenderink, van Doorn, & Lappin, 2000, 2003; Musatov, 1976; Schoumans, Kappers, & Koenderink, 2000; Todd, Oomes, Koenderink, & Kappers, 2001; Wagner, 1985). The concept of a curved visual space results from the integration of parallel and distance alleys in one space. The integration may not be allowed because parallelism and equal size may concern different spaces. The parallel alleys are parallel in visual space, not in physical space. The distance alleys are based on the size–distance invariance hypothesis, a mechanism causing that equally large objects positioned at different distances in physical space (Figure 1(c)) are perceived as equally large, although the objects are unequal of size in visual space (Figure 1(d)). Thus, parallel alleys reflect a special condition in visual space and distance alleys may reflect a special condition in physical space.

Carlson (1960) identified initially three and later four (Carlson, 1962) classes of instruction that affect size judgments considerably. The instructions were called objective, perspective, apparent, and projective. Effects of instruction were confirmed in other studies (Epstein, 1963; Gilinsky, 1955; Leibowitz & Harvey, 1967, 1969). Perspective and objective instructions cause overestimation of size with distance. Overestimation may reflect overcompensation of differences between representations of visual and physical space. Negative distances of the vanishing point simulate such overestimations in the perspective-space model (Figure 4). Apparent instructions caused underestimation if the instruction was given first and resulted in almost perfect size estimation if the instruction was given after judgments under perspective and objective instructions (Carlson, 1962). The apparent instructions may cause size judgments to occur in representations of either visual or physical space. The projective instruction causes strong underconstancy, where the size judgments seem governed by retinal size. Similar judgments occur under reduced cue conditions (Thouless, 1931; Holway & Boring, 1941). It may indicate that observers, at least up to a certain extent, have access to their retinal images. Retinal access is associated with a type of visual perception called proximal perception (Todorović, 2002). Proximal perception is controversial already for a long time (Hastorf, 1950; Hochberg & Hochberg, 1952; Ittelson, 1951) and still is today. Recent studies question proximal perception in laymen as well as artists (Perdreaux & Cavanagh, 2011, 2013).

## Conclusion

Perspective space is a simple, intuitive, and powerful model of visual space. It is simple because a single parameter defines its geometry. It is intuitive because perspective space is a trade-off between physical space and a two-dimensional projection of physical space representing the retinal image. It is powerful because it describes experimental results, explains visual phenomena and unifies a number of models of distance and size perception.

## Appendix

### Derivation of Equations (1) and (3)

In Figure 3(a), the line (black, dashed) connecting *S_p_* to the observer is described by: $x_{1}=\frac{S_{p}}{Z_{p}}z$.

The perspective line (red, dashed) is described by $x_{2}=S_{p}-\frac{S_{p}}{\mathrm{VP}}z$.

At the intersection, $x_{1}=x_{2}=S_{v}$ and $z=Z_{v}$ from which it follows that $\frac{S_{p}}{Z_{p}}Z_{v}=S_{p}-\frac{S_{p}}{\mathrm{VP}}Z_{v}$ which can be simplified to $\frac{Z_{v}}{Z_{p}}+\frac{Z_{v}}{\mathrm{VP}}=1$. Rearranging the equation gives Equation (1).

In Figure 3(b), Equation (1) yields for both the standard and comparison stimulus so that: $\frac{S_{\mathrm{vs}}}{S_{\mathrm{ps}}}=\frac{\mathrm{VP}}{Z_{\mathrm{ps}}+\mathrm{VP}}$ and $\frac{S_{\mathrm{pc}}}{S_{\mathrm{vc}}}=\frac{Z_{\mathrm{pc}}+\mathrm{VP}}{\mathrm{VP}}$. Multiplication of left and right sides of the equations with each other gives: $\frac{S_{\mathrm{vs}}}{S_{\mathrm{ps}}}\frac{S_{\mathrm{pc}}}{S_{\mathrm{vc}}}=\frac{\mathrm{VP}}{Z_{\mathrm{ps}}+\mathrm{VP}}\frac{Z_{\mathrm{pc}}+\mathrm{VP}}{\mathrm{VP}}=\frac{Z_{\mathrm{pc}}+\mathrm{VP}}{Z_{\mathrm{ps}}+\mathrm{VP}}$ or $\frac{S_{\mathrm{pc}}}{S_{\mathrm{ps}}}=\frac{S_{\mathrm{vc}}}{S_{\mathrm{vs}}}\frac{Z_{\mathrm{pc}}+\mathrm{VP}}{Z_{\mathrm{ps}}+\mathrm{VP}}$, which equals Equation (3).

### Derivation of Ooi and He’s Equation for Fixation of the Object on the Ground

Figure 9 shows that $Z_{p}=D/\text{cos}\alpha$. Furthermore h=dcosη and $h=Z_{v}\text{cos}\alpha$, from which it follows that $Z_{v}=\text{dcos}\eta /\text{cos}\alpha$

According to Equation (1) $Z_{v}=\frac{\mathrm{VP}}{Z_{p}+\mathrm{VP}}Z_{p}$, where *Z_v_*, *Z_p_*, and *VP* are distances in the viewing direction.

Substituting *Z_p_* and *Z_v_* in the expressions for *d, D, α,* and *η* gives $d\frac{\text{cos}\eta }{\text{cos}\alpha }=\frac{\mathrm{VP}}{\text{D}/\text{cos}\alpha +\mathrm{VP}\text{cos}\alpha }\frac{D}{\text{cos}\alpha }$ or

$d\text{cos}\eta =\frac{\mathrm{VP}\text{cos}\alpha }{D+\mathrm{VP}\text{cos}\alpha }D$.

The term VPcosα is the horizontal component of *VP*. The perceived ground plane is directed to the horizontal component of the vanishing point and, therefore, VPcosα=H/sinη.

Substituting H/sinη for VPcosα in the equation of dcosη gives $d\text{cos}\eta =\frac{\mathrm{DH}/\text{sin}\eta }{H/\text{sin}\eta +D}$.

From this equation it follows that $d=\frac{\mathrm{DH}/\text{sin}\eta }{H\text{cos}\eta /\text{sin}\eta +D\text{cos}\eta }$. The latter equation is identical to Equation (7).
